# Workplace Experiences of Women With Disability in Sport Organizations

**DOI:** 10.3389/fspor.2022.792703

**Published:** 2022-01-27

**Authors:** Clare Hanlon, Tracy Taylor

**Affiliations:** Institute of Health and Sport, Victoria University, Melbourne, VIC, Australia

**Keywords:** employment, intersectionality, women, disability, sport

## Abstract

Women with disability often experience barriers to employment and career progression, most notably in hyper-masculinised industry sectors such as sport. Applying an intersectionality lens and insights from critical disability theory, this research explores the lived experiences of eight women with varying types of disability through their stories of working and volunteering in sport organizations in Victoria (Australia). Analyses of the interviews highlighted the importance that these women attached to their identity as a woman with disability and the intrapersonal and organizational factors that impacted on initial sport workplace attraction and retention. The findings discuss the relationship management strategies adopted to manage these factors in workplace interactions. The interactive effect between disability and gender contributes to building a meaningful understanding of the intersectionality for women with disability in sport organizations.

## Introduction

Much has been written about how (mainly western) sport organizations, across countries and cultures, have historically been dominated by heterosexual, able-bodied white men who have shaped organizational culture and controlled decision-making (Knoppers, 2015; Burton and Leberman, 2017; Vianden and Gregg, 2017; Hancock et al., 2018). The masculisation of sport has manifested in the marginalization of many sub-groups along gender, age, racial, ability, and socio-cultural domains. Collectively studies demonstrate that sport leaders are a relatively homogenous, privileged group (Evans and Pfister, 2020). In examining the demographic composition of those in leadership positions, research has largely focused on the number of “minority” board members or in leadership positions (Adriaanse, 2017; Sisjord et al., 2017; Pike et al., 2018; Wicker and Kerwin, 2020). The built-in assumption of much of this work is that attaining gender equity in leadership positions will lead to more inclusive and diverse sport organizations (Hovden et al., 2018). In support of this proposition, a recent Australian study found “strong connections between gender composition of leadership teams and the reinforcement of gender norms and the level of progression of women” in sport organizations (Banu-Lawrence et al., 2020, p. 577).

Research agendas are starting to broaden and consider women's representation in other types of sport employment (cf Joseph and Anderson, 2016), including as coaches (de Hann and Knoppers, 2020) and professional athletes (Taylor et al., 2020). However, studies of general workplace participation at “all” levels of sport organizations, and the intersection of gender with other inequities, such as ableness, sexuality, and ethnicity, are sparse (Knoppers et al., 2018).

Careers for women in sport are shaped by individual and organizational factors. Women's lived experiences in sport organizations have been found to be constrained by: work-life integration challenges and a reluctance to pursue leadership roles (individual); and a lack of mentors, role models, networks, recognition, opportunities, resources, and policies that support positive work-life balance and instill conscious and unconscious bias (organizational) (Chisholm-Burns et al., 2017). In their systematic narrative review of women in sports leadership research, Evans and Pfister (2020) observed that many meso-level studies have investigated discursive constructions of gender, gendered disciplinary practices and organizational level gendered discourses (e.g., Claringbould and Knoppers, 2012), but noted that investigation at the micro-level is less common. Research has found that women face significant barriers in the attainment of leadership positions in Australian sport (Gacka, 2017; Richards et al., 2020). Limited research (Joseph and Anderson, 2016; Clayton-Hathway and Manfredi, 2017; Darcy et al., 2017) explores the nexus of gender and disability in sport leadership or sport organization employees, although some work points to those in positions of leadership in sports clubs (the in-group) resisting any change to their privilege, especially in relation to increasing diversity (Spaaij et al., 2020). Research has also revealed that people with disability are often afforded “second-class” status in sport due to a systemic and internalized ableism (Kearney et al., 2019).

In general terms people with a disability experience lower employment rates, and are afforded fewer opportunities for management and leadership roles (Roulstone and Williams, 2014; Plowman, 2018). This inequity in opportunity has been associated with employer perceptions that hiring people with a disability incurs additional costs and challenges (Waterhouse et al., 2010), which in turn creates anxiety about appointing people with disability (Domzal et al., 2008). There is little research into sport organizations' employment of people with disability (Wright and Cunningham, 2017). Research about sport and people with disabilities has largely centered on the experiences of this cohort as recreational participants or high performance athletes (Misener and Darcy, 2014; Darcy et al., 2017), exploring how individual, societal and organizational structures, perpetuate systemic exclusion, prejudice, and stigmatization (Dane-Staples et al., 2013; Kitchin and Howe, 2014). Notably, Roulstone and Williams (2014) highlighted the need for further research which enhances our understanding of the experience of people with a disability through identifying the factors that influence how impairment and disability status is managed in the workplace and strategies used to negotiate key relationships. The development of trusting in these relationships is critical, with Shantz et al. (2018) finding that while having a formal disability policy is important, combining this supportive human resources management practices leads to greater job satisfaction for employees with disability. Negotiating relationships can take on many forms, with “surface-acting” being one way that people with disability manage the expression of their internal feelings to display an emotion different to what is being felt (Hochschild, 1993). The visibly projected emotion is usually more positive that what is actually being felt (Mann, 2005). Boucher (2017) noted this is akin to women with disability actively managing in ways to “disappear” their disability in the quest to fit into the workplace.

In the quest to bring together gender and disability; the purpose of this study is to address the lack of research on the intersectionality of women with disability and their lived experiences of working and volunteering in sport organizations. The social model of disability, located within the complexities of intersectionality, guided the formation of our two research questions, “how do women with disability experience constructed difference in the sport workplace?” and “what are the organizational responses perceived by women with disability on their requirements and career expectations?”

## Literature Review

### Australian Context

There is limited data on people with disability working in the Australian sport industry, and few, if any, published studies on their experiences, The federal legislation, Disability Discrimination Act 1992 p. 19 (Australian Government, 2016), notes it is “unlawful for an employer or a person acting or purposing to act on behalf of an employer to discriminate against a person on the ground of the other person's disability,” related to the arrangements made for the purpose of who should be offered employment, who should be offered employment, and the terms or conditions on which employment is offered. Once a person with disability is employed, it is unlawful to discriminate against an employee on the ground of the employee's disability in the terms or conditions of employment, denying employee access or limiting the employee's access to opportunity for promotion, transfer or training or any other benefits associated with employment, by dismissing the employee, or by subjecting the employee to any other detriment (Section Limitations). The Act specifically references sport noting, it is unlawful for a person to discriminate against another person on the ground of the other person's disability by excluding that other person from a sporting activity (p. 29), this would include, but does not specifically mention, volunteering.

In Australia, 48 per cent of working-age (aged 15–64 years) people with disability are employed with women having a lower employment rate than men (46% compared to 50%) (Australian Institute of Health Welfare, 2020). Data on women as leaders in the sport industry is scant, women comprise 14 per cent of coach, official, and/or administrator/committee member roles comprise (Sport Australia, 2021). No data exists on the proportion of leaders who are women with disability in sport. The lack of information on women with disability who work and/or volunteer in sport reinforces the need to gain insights in order to build the understanding of employers on good practices and strategies to encourage women with disability to roles in sport. Furthermore, it has been argued that research which gains insights from people with disability on their experiences, regardless of sector (Williams and Mavin, 2012), is a deficit in organizational studies. Williams and Mavin argue that to address this gap, theory should be inclusive of people with disability, through the use of a disability studies lens which considers how they strategise and negotiate social contexts.

### Studying the Nexus of Gender and Disability

We explore the lived experiences of women with varying disability by drawing on broader insights from disability and organization studies to provide insights into “otherness,” workplace relationships and networks, collective identity, and inclusion. Otherness refers to individuals “marked with the seal of difference, be it physical or bodily (color, race, handicap, gender, etc.), moral (lifestyle, sexual orientation) or linked to the membership of a particular group (national, ethnic, communitarian, religious, etc.), stand out in social or cultural entities” (Jodelet, 2005, p. 26). It is a perception of the other belonging to a category that is quite separate from one's own and thereby directly refers to social identity, where people consciously believe themselves to be members of a particular social group (Harma et al., 2013). While some people with disability may be politically, economically, and socially integrated in society, they however continue to be identified in the otherness category (Harma et al., 2013). This otherness is located within a framework of discursively constructing disability in relation to normative perceptions of non-disability (ableism) through which the organizing experiences of people with disability are viewed (Williams and Mavin, 2012).

We recognize that the disability community is not homogenous and that it comprises a diverse group of people who “experience impairment and societal discrimination on the basis of that impairment” (Forber-Pratt et al., 2018, p. 2). The disability community encompasses many intersectional identities, and represents people with a range of disabilities and experiences of impairments. Disability identity relates to how a person takes pride or feels shame of their disability, and the engagement, comraderies and contact a person has with the larger disability community (Caldwell, 2011). In the present research we sought to gain insights from, and take account the disability identity of women with disability working or volunteering in the sport sector.

### Critical Disability Theory

The social model of disability guided this research by providing a theoretical and practical framework to explore and address concerns related to disabled people (Barnes, 2012), with a focus on social and environmental barriers that create disablement and disability. Disability discourses are conceptualized within a socio-political context, emphasizing the (disabling) role of societal structures and environments in the experiences of people with disability, and simultaneously addressing how people with disability continue to be marginalized (Charmaz, 1995; Barnes and Mercer, 2004; Forber-Pratt et al., 2018). The model acknowledges and legitimizes the lived experience of a person with disability. The consideration of individuals' life stories is essential to understanding the complex interplay between impairment, disability and environment, and hence, how disability is experienced (Darcy et al., 2017).

Central to the understanding of the social model of disability is the consideration of the individual and their impairment within the socially constructed disabling environments. It is personal experiences, the social construction of the environments in which people live and work, and social reactions that “disable” people (Purtell, 2013). The social approach recognizes inequities experienced by people with disability are a result of ableist social and environmental factors (O'Connell et al., 2008). The social construction of disability (Meekosha and Shuttleworth, 2009) notes the complex interplay of social power dynamics, normalization, inclusion/exclusion, accessibility, mobility, identity politics, intersectionality and privilege (Titchkowsky, 2011). Understanding the impacts of these interactions can be achieved through identifying socio-structural features and the social contexts that influence how people with disability negotiate their environments (Chalk, 2016).

While acknowledging the fundamental premise of the centrality of social construction of lived experience in the social approach to disability, Brewer et al. (2012) have argued that disability can be also be viewed as a positive identity and forge “a positive definition of disability identity based on experiences and circumstances that have created a recognizable minority group called “people with disabilities” (p. 5). Social identity can be formed through association with one or multiple personal or group characteristics. Recent research has found disability social identity, that is forming a collective identity within a marginalized group, such as being a person with disability, can be a positive psychological resource for well-being (Bogart et al., 2018) and psychological health (Bogart, 2014). People who have strong disability identities can challenge societal, physical and systematic barriers that have made life with a disability challenging (Forber-Pratt et al., 2018). In a study of female managers with disability in the workplace, Boucher (2017) noted that when these women found themselves at the intersection of their (i) leadership position, (ii) gender and (iii) disability, the three-prong multiplier effect often necessitated actions that minimized the visibility of their disability. In doing so, two contingency factors evolved: interpersonal and organizational that when combined created strategies these women leaders with disability used to manage their workplace relationships (Boucher, 2017).

### Intersectionality

Originating in critical race feminist writings on the marginalizing experiences of Black women, the term intersectionality was first used by Crenshaw (1991) as an analytical lens for situating Black feminist critique of experiences of gender and race. Intersectionality theorizing has since expanded to include a broader range of social groupings including disability, to gauge how these interact in different ways to create marginalization or social exclusion (Collins, 2000). Intersectionality has also been used to demonstrate compounding effects of multiple identities, both in terms of advantage or disadvantage (Emmett and Alant, 2006) and as an analytical tool to capture and engage contextual dynamics of power (Cho et al., 2013).

In a call for greater use of intersectionality sensitive approaches when studying employment relations and work, McBride et al. (2015) argue the importance of engaging with the transecting experiences of those who fall into different categories of marginalization. This means including research agendas that explore the intersection of such categories (e.g., gender and disability) and that question the relationship between categories of difference. McBride et al. (2015) suggest that complexity in the interpretations of intersectionality theory have held back a more extensive application beyond critical feminist scholarship. Intersectionality theorizing provides a basis to explore the experiences of people who have more than one category of social differentiation (i.e., women with disability). The use of case studies to study intersectionality can be used to identify the complexities and differences of experience by the user group (McCall, 2005). Complexity is derived from the “analysis of a social location at the intersection of single dimensions of multiple categories” (p. 1781), such as the workplace and the employee/volunteer, such as women with disability. Furthermore, as McCall notes case studies can “uncover the differences and complexities of experience” (p. 1782). Single-group studies that comprise personal narratives are of interest in capturing social relation insights to the identity of particular social groups (McCall, 2005).

To address the silencing of our knowledge about women with disability who work and/or volunteer in sport, located within the complexities of intersectionality, the social model of disability guided the formation of our two research questions. Our research contributes to broader research agendas that include people who have been theoretically and organizationally marginalized (Williams and Mavin, 2012).

## Method

The methodological approach was informed by McCall's (2005) call for single-group case studies that comprise personal narratives. This taking this perspective we argue that complexity derives from exploring the experiences of women with disability at the intersection of single dimensions of multiple categories, noting the social relations in the identities of these women within the sporting context. To address the research questions, it was critical to gain nuanced narratives of women's experiences as employees and volunteers in sports organizations. In doing so identify what implications this has for sport organizations to adopt in building inclusive practices. This was achieved through the use of semi-structured interviews that enabled the exploration of discourses of organization practices experienced by women with disability.

The project was applied in nature and researchers worked closely with a collaborating disability and sport industry partner organization to identify and access study participants. Participant criteria included women (aged 18+ years), who were employed or held a voluntary leadership role within sport in Victoria (Australia). Our collaborating partner drew on their industry contact database to identify these women and 20 women who fit the criteria were contacted about expressions of interest. The women who volunteered to participate were provided additional information about the research and the interview protocols which followed university approved ethics guidelines. After conducting eight interviews it was determined that thematic saturation was reached (Minichiello et al., 2008).

All women were from a western background, the majority (5) were aged between 20 and 29 years. Half of the participants held paid roles in sporting organizations, and seven of the eight women held leadership positions in sport for an average of 4 years. Two women identified as having a physical disability, three had a visual disability and three had hearing impairments. In addition to working in sport, every woman played sport. A demographic summation of interviewees is presented in Table 1.

**Table 1:** Demographics of interviewees.

**Pseudonym**	**Role**	**Paid/Voluntary**	**Yrs in role**	**Disability**	**Age**	**Sport played**
Jill	Non-executive Director	Voluntary	5	Vision impairment	20–29	Down-hill skiing
Kate	Volunteer coordinator	Paid	3	Hearing impairment	20–29	Tennis
Larissa	Administration	Paid	4	Physical impairment	20–29	Wheelchair basketball
Malina	Board member, Club secretary	Voluntary	14	Hearing impairment	30–39	Cricket
Sharon	Marketing coordinator	Voluntary	4	Hearing impairment	40–49	Cricket
Talia	Regional school coordinator	Paid	1	Vision impairment	20–29	Goalball
Tara	State Association Secretary	Voluntary	2	Vision impairment	30–39	Goalball
Yolanda	Program leader	Paid	3	Physical impairment	20–29	Wheelchair football

The semi-structured interview guide was based on literature on disability identity and gender intersectionality, with particular emphasis on the socio-structural features that influence how women with disability negotiate their work environment (Chalk, 2016). To assist these women feel comfortable during the interview, the questions were emailed at the same time as the Information to Participants consent form. In addition, interview support was offered and in consequence an interpreter was employed for the three women with hearing impairment. In the interviews, women were asked about their personal experiences of sport employment/volunteering, their perceptions and experiences of difference or marginalization, specific actions, practices or policies that either facilitated or prevented inclusion.

Interviews ranged from 50 to 90 mins, were audio-recorded and transcribed verbatim with the permission of the participants. Transcripts were coded using NVivo 12 through a thematic analysis process of identifying, analysing and reporting patterns (themes) and domains (categories). While recognizing feminist critiques of the artificiality of social categories, we suggest social reality of categorisation in empirical studies of intersectionality can beneficially explore finer intersections of categories by taking an intra-categorical approach to complexity. Taking this approach, each researcher (the authors) and an external coder, read the transcripts to identify themes, and this was followed by a process of constant comparison until agreement was reached as a check for intercoder reliability (Miles et al., 2014). Systematic coding enables richness, nuance and consensus in the representation of participants' experiences.

The iterative coding process ascribed deductive codes to “units of meaning” such as portions of the text according to the process outlined by Campbell et al. (2013). Coding according to units of meaning was appropriate due to the flowing nature of the conversation between the interviewer and participant. A deductive process was used to identify the ways in which women with disability negotiated work environments and managed workplace relationships, these were categorized into overarching domains aligned with the contingency approach (Boucher, 2017). Given the researcher's centrality in coding, verification strategies included sample participant relevancy, and a familiarization process was incorporated that involved multiple iterations of coding and thinking theoretically to support the trustworthiness of the data analysis (Campbell et al., 2013). The researchers collaboratively reviewed their interpretations of the interview data, clarified inconsistencies, and explored alternate explanations to further reliability. Crucial to maintaining rigor in the analytic process was engagement with the interviewees to facilitate trustworthiness, and act as critical members of the research process. All interviewees whose “voices” appear in this study were consulted to check that their statements were accurately reported and not presented out of context. Through this process, we were able identify 10 themes together with the two domains. We discuss each of these in the next section.

## Results

**Figure 1:**
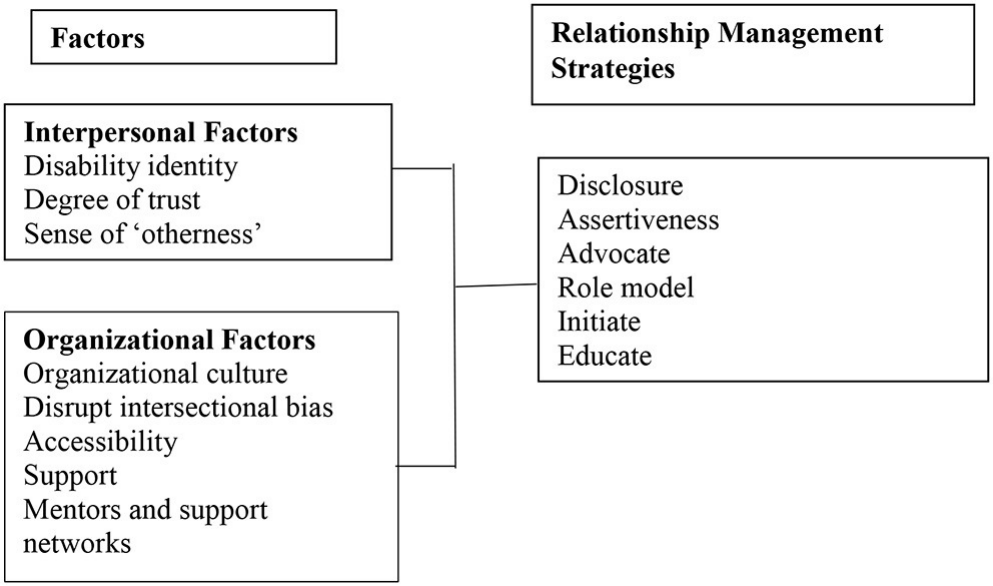
Relationship management strategies adopted by women with disability.

The positive attributes associated with sport were central to every participant's decision to seek work and/or volunteer at sport organizations. Sport was perceived as an enabler with a positive culture. On entering the workplace, these positive attributes were at times found to be more rhetoric than reality. A common response was to take a contingency approach to remaining in the organization, underpinned by intrapersonal and organizational factors (see Figure 1). The overarching domains of interpersonal and organizational aligned with Boucher's (2017) two factor contingency approach for categorizing workplace relationship management. The interviewees disclosed various strategies that they adopted to manage these often intersecting factors when interacting with others in the workplace. Interpersonal factors identified were classified as interactions with managers and employees; organizational factors encompassed the culture, commitment and provision of services, or lack thereof, that combined to present an inclusive or exclusionary workplace environment for women with disability.

### Sport as an Enabler

The interviewees spoke about their positive experiences playing sport and were generally passionate about what sport provides to individuals and the community. Key attributes that sport builds included: confidence [it] “helped me with getting comfortable in my own skin” (Yolanda); the ability to challenge oneself; empowerment based on achieving goals; social engagement; and a healthy lifestyle that “saved my life” (Larissa). The general perception was “you can usually pick the people who play sport from the people who don't because of the way they conduct themselves, their confidence, social skills and the way they go about their lives” (Tara).

Sport was also perceived as enabling a positive community culture through: fun; people working together to achieve a common goal; providing role models; and building strong supportive communities that make a “difference to people's lives especially when it comes to having a disability” (Yolanda). As a result of playing sport these women wanted to be part of the sport community and create community change for a healthy lifestyle, provide other people with the same opportunities they experienced, and to give back to the community. However, on entering the sport organizations as an employee and/or volunteer they found that different mind-set was required including developing strategies to manage interactions with others to feel included in the workplace.

### Interpersonal Factors

The three interpersonal factors that emerged from the analysis were their *identity* as a woman with a disability, the degree of *trust* these women have with their manager and team around them, and the need to lessen the sense of “*otherness.”* These factors were important to gain empowerment, build confidence and to become a team member in the workplace.

All of the women interviewed were explicit about the centrality of their *disability identity* in their employment situations. Given the low number of employees with a disability in the sport sector, they strongly believed that full disclosure of their disability was critical in highlighting that women with disability were highly capable employees and volunteers. This assisted in breaking down stereotypes and expectations of the stereotype employee, described as “blonde, tall, athletic, white women who are high achievers” (Talia). They spoke about the process of initiating immediate disclosure of their disability to their employer, based on past “trial-and-error” experiences where non-disclosure created issues later on. Even in cases where it was stressful to do so, revealing their disability provided a starting point to establish ways to address employer questions or make required accommodations. One woman proudly stated:

I've decided to disclose every single time. My disability is a very big part of who I am and it is about education for everyone all the time. So, that is it… while it might make me uncomfortable (Malina).

Several of the women indicated that their inclusion was most effectively facilitated through strategies that recognized and valued their disability identity, and when others in the workplace were open-minded and curious about their needs. This involved asking questions with a positive and accommodating mindset, “number one is to make sure that you're open-minded, to be adaptable to the needs of someone like me.” (Talia).

Women who had successfully navigated various forms of exclusion and discrimination, and who openly displayed their disability identity, were approached by organizations “asking for help to draft a new terms of reference or best practize documents…. [to address gender and disability matters] (Jill).

The *degree of trust* these women had in their manager and those around them in the workplace impacted on their confidence in exactly what to disclose about their disability. Even if their disability was less visible or obvious, when they felt confident in their workplace environment the women then broached their disability and associated adjustments required. Being comfortable in initiating the conversation was empowering:

The day I realized I could take that power into my own control was when I realized I should actually start conversation rather than have it dictated to me. That's where I gained that inner confidence where I became confident enough to say, “Hey, this is my situation” (Kate).

Relational trust is underpinned by an assumption of acting in good faith, and having trust in management (Shantz et al., 2018). Building of relational trust can lead to the development of institutional trust, that is, where the sport organization is trusted to enact and abide by its policies on inclusion and equity. The women who had developed trust in management, derived from repeated, positive interactions in the workplace, expressed a sense of belonging and support. Cultivating a relationship is an example of establishing relational trust, in the case Larissa it was with her manager, “my manager was always checking in when a decision needed to be made about resources I may need, she was always asking the right questions like “Is this going to be okay, and what about this? What about your desk? What does that look like?”

In some cases experiencing a *sense of “otherness”* meant these women felt conscious that their gender together with their disability hindered them from being an “equal” member of the team in the workplace. In these instances, women did not feel comfortable to openly disclose their disability and workplace needs. Productive relationships with managers and work colleagues were significant to whether feelings of otherness persisted or diminished over time. Open and honest interpersonal relationships acted to facilitate a greater sense of inclusion. Approaches which developed positive experiences with senior leaders in the organization, and being upfront about the lack of knowledge about a disability created enabling experiences: “I think this is the most inclusive workplace that I've worked at because as soon as I started my CEO approached me and said straight up “we've never worked with deaf people before and we're really happy to have you here, how can we help you?” (Talia).

The interviewees spoke positively about the importance of workplace colleagues that acknowledge their “otherness,” where the woman and the disability intersect, to conjointly value the individual as an employee first and foremost. On the other hand, some interviewees expressed concerns that their managers did not think that women with disability held leadership aspirations. To address this issue, these women indicated a successful strategy was to initiate conversations and make their intentions clear, as described by Kate:

Talk to people, let people know that you have aspirations for development because they will champion you and if they see a scholarship or an opportunity and know that you're interested, then they're going to put you forward.

Interactions which de-bunked or challenged social categories of difference, contested an assumed privileged position of ableism. To be valued for who they were with experiences and positionality meant these women felt *distinct* rather than *different*. It is about recognizing the person and their distinct needs, “support deaf women and support us to grow” (Talia) by meeting specific workplace adjustments or requirements.

### Organizational Factors

Five, often intersecting, organizational factors emerged from the interview data analysis as workplace based considerations and provisions in the attraction, retention and career progression of women with disability. These included: organizational culture; disrupt intersectional bias; accessibility; support; and mentors and networks.

*Organizational culture* was identified as a distinct factor, but it should be noted that an organization's approach to the other four factors clearly influences its broader workplace culture. The importance of an inclusive organizational culture for women with disability was voiced by all, as an inclusive culture created a welcoming environment. Larissa shared her positive experience “It was great to be exposed to like-minded people that love the sport, that want to make it better, include everyone and want to see it succeed from grassroots to elite competition.” Workplace acceptance and confidence was built through the collective organizational attitude and behaviors of individuals, team members and managers. An inclusive culture was characterized by the absence of judgement about, or negative responses to being a woman with disability, and was of particular note when working in traditionally male dominated sports with few women employees. As one interviewee observed “male dominated sports can be very competitive, very oblivious to anyone else except themselves and consequently that value systems is driving all sorts of decisions” (Malina). Asking questions was seen as a key strategy in addressing discrimination, and was important in making women feel comfortable in their work environment:

Have an open mind and ask questions. Every person you come across, every female you come across with a disability, even if they have diagnosed the same disability are still going to have different needs and if you ask them, build that rapport and have that open communication, that's what's going to help us feel comfortable (Tara).

Organizations with inclusive cultures were depicted as initiating consultation, whereas less developed inclusive cultures were characterized as needing to be prompted:

People generally want to do the right thing 99% of the time, they just don't know how. It is half my responsibility to show them how. Half their responsibility to listen (Sharon).

The interviewees stressed that *disrupting intersectional bias* of gender and disability, was essential on providing an inclusive organizational culture. These women were conscious that their intentionality was perceived as a barrier: “people seeing the fact that you have a disability or that at the high end [of management] is not where women belong, [the sport] is very heavily dominated by men and to get there is really challenging” (Malina). The need to advocate and educate work colleagues were actions taken by these women to negotiate situations that served to disrupt intersectional bias. In doing so, they indirectly became role models for other women and/or people with disability. Based on her lived experience, Kate was focused on gaining director positions to disrupt intersectional bias, “There are a lot of limitations and barriers in place for minority groups and women and people with disability both fall into these. When you combine them, that's an extra layer of barrier and for me the desire to pursue governance skills and directorships has been through wanting to be part of ensuring the people on the ground have the ability to have their voices heard.”

Intersectionality in the workplace was identified by Kate from a three-fold perspective: age, gender and disability. As a result, Kate initiated and sought external mentoring and developed internal relationships:

The board that I sit on, I'm the youngest by a long way and I'm also a female and I also have a disability so tick a few minority boxes in the directorship space. Finding a mentor that I could talk to externally and create relationships with those in the organizations has been really helpful.

The women interviewed were passionate about advocating for formal inclusion training as an essential initiative in the workplace. As a result of their actions, some interviewees reported that after the training was implemented managers and work colleagues began to understand and see the capability of the individual before the disability: “It's about seeing the person and what they can contribute and being willing to put that support in place to allow people to thrive” (Yolanda). Training that has been successful, as perceived by Malina, involves all staff regardless of level, to understand “what inclusion means. What inclusion and disability and diversity looks like… How you should treat someone and just the day-to-day things.”

Awareness and experience of societal and organizational challenges women with a disability face and strive to overcome to have a successful career were presented by the interviewees as evidence of drive and fortitude. “Females before us have spent a lot of time trying to equalize the opportunities within the work environment. If you have employed a female with a disability, because of the history in employment, they're going to be quite driven” (Talia).

*Accessibility* related to resources, adjustments in the workplace and creating physical accessibility, was unsurprisingly, identified as a critical organizational requirement. In regards to sport organizations, “Accessibility is really important for sporting organizations to understand, implement and change the narrative around understanding of accessibility because it's not that hard to do and implement” (Jill). Women's accessibility requirements varied according to the nature and extent of their disability. Strong sentiments of acceptance, support and feeling valued by their employer were associated with organizations accommodating accessibility requirements, taking requests seriously and prompt action:

With my current work, I needed more computer support and it was seamless, it wasn't a big deal… Whereas previous employers are like “Oh yeah” and then ages later, I'm like, “Remember we had that conversation?” So I think the understanding, if a request is asked in terms of disability it's actually a necessity, not just a request (Tara).

An organization's ability to provide appropriate accessibility responses meant being aware of position selection criteria that are inherently exclusionary, such as requiring a driver's license. Many sport organizations have this obstacle to employment in place based on the need to travel to collaborate with sport clubs. As a result “I find that as soon as I tell people that I don't have access to a car, but I can get around other ways, that's a barrier and they won't budge” (Talia). Improving accessibility can be achieved in many ways, such as electronic distribution of documents for employees with a visual impairment. The way in which the organization facilitated appropriate access was taken as a proxy indicator of an inclusion culture. For example, there was an expectation of at least full adherence to legislated Australian Standards for access to premises/ buildings (Australian Government, 2010). However, this was not always the case and when absent a sense of surface-acting was portrayed where women questioned whether the employer had considered how to facilitate workplace inclusion: “like not having Braille on lifts… It's really simple stuff like that, that's saying at an implied level, you're not welcome here. We'll not accommodate, we haven't planned for you, because we didn't expect you to be here” (Jill).

As organizations were not always cognisant about the best way to make adjustments in the workplace to enhance accessibility, the need for ensuring that the organization has a strategy of genuine consultation and communication was highlighted: “If you don't consult with people with actual disabilities [with regard to] what makes your workplace accessible and inclusive, and you just go and do all these things that you think are really good, [you will find] they don't actually work in practize” (Jill). The interviewees suggested that developing action plans that enable sport organizations to grow with a diverse workforce were useful in facilitating adjustment. Having plans that recognize the important role women with disability provide and their capacity was stressed as a critical element, “I've even been involved in training sessions at [sport] where they were keen to gain my thoughts” (Yolanda).

Sport organizations that embedded accessibility considerations into recruitment, induction, and the everyday work environment were also more positively viewed in terms of having an inclusive organizational culture. Strategies to go beyond the “surface level” response included providing clear information on parking, accessibility requirements and on the employment role. In illustration, during the induction stage being asked questions about how to address any accessibility issues:

I think like any individual who's being inducted for a job, the process is individualized to their needs and their role and capacity. I would suggest that the exact same thing happens. If there are just the considerations for that individual's needs and so in this case if it's a female with a disability, then perhaps it's talking about: how can we make this information accessible to you? Or how can we make this facility accessible to you? (Kate)

Although there were many positive stories, most participants described situations where their request for access resulted in a negative experience. As a result, they felt unsupported and left that particular organization:

My last position, they didn't offer any parking. The street parking has restrictions everywhere. I had to move my car, and on some days, wasn't able to, and so I flagged it and said, “This is not accessible,” and they said, “Well, you'll have to pay for undercover parking.” I said, “Well that's not accessible either.” So that was like a big issue, and in the end I just said, “Look, it's not worth my time if just getting here prohibits me from being the best version of myself.” And they weren't willing to budge, even though I was on the disability working group (Malina).

Negative accessibility experiences prompted several of the women interviewed to assume an advocacy role for people with a disability, working to educate organizations. Jill's negative experiences strengthened in her resolve to become a sport board director, “when you're the bottom of the governance hierarchy there are a lot of limitations and barriers in place, in particular for minority groups and women and people with disabilities fall into those.” Relationship management actions were also taken and included being assertive in situations that were not satisfactory, such as when reasonable adjustments were not forthcoming. Advocacy organizations acted as a helpful resource for women with disability to drawn on for support in facilitating difficult communications:

…advocacy organizations are so important for people who have intellectual disability or something like that, who just don't have that capacity to go, no, this is wrong. This is outside the scope of my role (Jill).

*Organizational support* from a human resource perspective was deemed important, starting with the first interaction with the organization. Support provided prior to job interviews was one common example provided by these women. Perceived support was gained when detailed information was provided on accessibility of the organization, clear directions to the interview room, and the allowance of time for women to arrange the support needed, such as interpreters. Jill described how being provided with this information gave her confidence: “you want to walk into the office knowing where you're going. You don't really want to come into the interviews, and say can you come and get [me at] the tram stop, please?” Access to the interview questions ahead of time was also seen as a useful strategy to assist women feel empowered during job interviews.

Organizational support in assisting with career development was a common theme in the interviews. Successful strategies that were highlighted included the explicit provision of opportunities for women with disability to attain and succeed in sport leadership positions assisted with their retention in the workplace. When support was absent and they were overlooked for progression opportunities and leadership programs, participants spoke about being having to be assertive and confident to put themselves forward for leadership roles:

My expertise right now is I've been a leader of basketball, a mentor, a coach and an elite player. These skills should be able to be transferred into a workplace, and it seems, based on my personal experience, that this wasn't even good enough for someone in the sports industry to say “We want you” (Larissa).

Access to ongoing professional development was viewed as an important component to assist women with disability build a career pathway in sport.

Having good *mentors and support networks* was identified by the interviewees as another key factor that supported their career success. Organizations that recognized the importance of facilitating access to appropriate mentors were viewed as supporting women with disability in their career journey: “mentoring gives somebody confidence. It makes them feel positive about themselves” (Talia). Mentors were sourced both externally and internally, assisting women with disability to navigate challenges and consider how to best manage, “relationships with staff within the organization” (Kate). Malina drew on her mentor as “someone who can guide you, in the business world but also in life… Someone who's done it before or has some insight into what are the little steps to get to the bigger goals.” Drawing their lived experiences as women with disability working in sport organizations, mentoring other women regardless of ability was identified as a way of assist to build more inclusive workplaces for women: “I hope I can be part of helping other women get into similar roles” (Yolanda). Mentors specifically supported the women interviewed but were not engaged in promoting disability issues more generally in these women's workplaces.

The extent to which they initiated or were involved in support networks varied among the interviewees. Several spoke about positive outcomes associated with the support of managers, work colleagues and mentors when these women were introduced into both internal and external support networks. Other participants initiated internal networks on their own and drew on the expertise of disability support and advocacy organizations for external support networks.

## Discussion

The confidence levels of women in this research shaped their intentionality in enacting relationship management strategies to address identified interpersonal and organizational factors to entering and progressing their careers in sport. Confidence to apply for a paid and/or even a voluntary role was based on their perception of reactions associated with intersectional bias of gender and disability in the sport sector, and in the organization's workplace culture more specifically. The intersectionality bias identified in this study is located within an inherently dominant masculine culture of Australian sport and entrenched entry barriers for women leaders (Richards et al., 2020), and further accentuated by sports' focus on ableism (Kearney et al., 2019). Ableism can be defined as a “system of oppression that faces disabled people in our society, a system that marks disabled people as inferior and most importantly, other” (Liebowitz, 2017, p. 153). In analyzing the women's lived experiences, the feelings of being identified in the otherness category (Harma et al., 2013) were clearly evident.

Confidence of being accepted into the workplace environment was built through experiencing an inclusive organization culture, seeing disrupted intersectional bias, having accessibility requirement met, and/or being provided with support and mentors and support networks. Findings from our research identified that having the confidence to be assertive and to put themselves forward in leadership roles and in securing a mentor, were seen as crucial to the career progression of women with disability. That these women were not “seen” or considered as having leadership aspirations by the organization's decision-makers reinforces Manfredi's (2017) contention that women need to tackle “invisible barriers” such as a gendered construction of leadership, accumulated disadvantage which is reinforced in persistent male-dominated cultures. Feeling less than confident in their own skills and abilities compared to men, is a consistent research finding particularly in the context of sport leadership (Fielding-Lloyd and Meân, 2011) and in sport organizations where “those who had experienced discrimination because of a disability were less likely to intend applying for promotion in the future than those had been discriminated against for other reasons, such as their age or gender” (Clayton-Hathway and Manfredi, 2017, p 42).

Having confidence enabled these women to instigate a workplace relationship management strategy appropriate for their employment context. The actions included the disclosure of their disability, to be assertive, and/or to advocate, initiate and educate managers and colleagues in the workplace. These strategies were used interchangeably across the interpersonal and organizational factors, such as being assertive and initiating conversations related to providing organizational support and accessibility requirements. The five relationship management tactics were “survival” strategies, deployed by these women with disability to negotiate their organizational environment. These are strategies found more broadly across sectors (Chalk, 2016) and are not specific to sport organizations.

Through their actions, the women interviewed who implemented relationship management strategies to influence interpersonal and organizational factors, fostered the development of role models. The women who disrupted the “norm,” forged a positive definition of disability identity (Brewer et al., 2012) through drawing on their experiences and circumstances. Subsequently, these women were sought as consultants by organizations to develop good workplace practices related to gender and disability. Having positive role models has been found to be an ideal way to encourage employability of minorities, especially when these role models are in visible senior level jobs in the workplace (Kurtulus and Tomaskovic-Devey, 2011). Incorporating good workplace practices from the lens of a women with disability assists organizations to address the lack of understanding employers have of disability employment issues (Waterhouse et al., 2010), employer anxiety about employing people with disabilities (Domzal et al., 2008), and has significantant implications for increasing the employment of people with disability (Boucher, 2017).

Our findings showed that the women interviewed felt that they were categorized by those in the workplace; placed in an “otherness category” (Harma et al., 2013). Otherness was associated with intersectional bias of being a woman, and a person with disability. These experiences can be located within the broader context of the persistent exclusionary male dominated culture of Australian sport organizations (Gacka, 2017). Gender, disability and, at times age, interacted as components associated with intersectional bias. To disrupt such biases in the workplace these women, in many instances, took it upon themselves to advocate, initiate, and educate managers, directors and work colleagues about diversity, inclusion and on the benefits of involving non-normative minority groups as employees and volunteers. Thus, creating transecting experiences for those who fall into the different categories of marginalization (McBride et al., 2015). Notably, the reported sense of “otherness” dissipated when these women were perceived as an “equal” member of the workforce. When intersectional bias was disrupted and they were valued for their knowledge, skills and abilities, these women felt the otherness category began to disappear and instead felt part of the team.

## Conclusion

This study contributions to the literature in several ways. First, in examining for the first time the experiences of women employees and volunteers with disability in the sport industry, we address calls to better understand diverse workplace experiences and give voice to those people and issues “silenced in meta-theoretical critiques in the study of difference in organization studies” (Williams and Mavin, 2012). In doing, so our findings complement other research showing that women with disability have more positive experiences in organizations with inclusive cultures and supportive human resource policies, and that disrupt intersectional bias (Boucher, 2017; Shantz et al., 2018).

Moreover, this research contributes through exploring both micro (interpersonal) and macro (organizational) factors and presents arguments that the individual's interpretation of being and feeling valued as a women with disability, means not being judged against norms of ableism, and trusting employers to provide supportive workplace adjustments as a matter of course rather than by exception. Micro processes and approaches that underpin organizational practices can act to marginalize women with disability, particularly where power and decision making is based on gender and disability is continuously reproduced (McBride et al., 2015). In looking at the experiences of women with disability from a social disability studies lens this study has shown how these women strategise and negotiate their social contexts (Williams and Mavin, 2012) at both the individual and organizational level.

We also contribute to the workplace disability literature which has shown that disability and gender considerations may require different human resource management approaches, but that while this is well recognized these needs are not necessarily addressed (Darcy et al., 2016; Shantz et al., 2018). Our findings regarding intersectionality also extend the literature in this area by showing the importance of deconstructing the nature of a disability, impairment and how this intersects with gender to shape one's articulation of work life (Jammaers and Williams, 2020).

### Implications for Practise

Numerous strategies that sport organizations and their employees could be more inclusive of women with disability were revealed in the findings of our study. Given the centrality of a welcoming and positive organizational culture and accessible physical environment, the implementation of employee education programs on the benefits of diversity and inclusion could assist with building an inclusive and positive organizational culture. Taking a collaborative design and delivery approach to training could assist with trust building and lessen the pressure on women with disability to assume an advocacy and educator role. A further consideration is to ensure that the requirements of women with disability are embedded in policies and practices covering recruitment, selection, induction, performance management and accessibility to the organization. In essence, the simple question: “how can we help you in this role?” carried great weight and meaning for our interview participants. It should be incumbent on every organization that employees are educated about, and living up to, basic inclusive workplace standards (Darcy et al., 2016).

A further consideration is to audit organizational human resource management practices against criteria for genuine and timely responses, and provision of access requirements to facilitate workplace inclusion. As previously noted, accessibility practices begin at the recruitment stage can be embedded into the everyday work environment. Such practices portray examples of committed leaders positioning the organization to create an inclusive environment (Waterhouse et al., 2010). Authentic inclusion negates the need for women with disability to engage in “surface acting.”

Our research identified two different types of relationship management strategies that were indicative of surface acting. First, managers were found to surface-act when agreeing to provide workplace adjustments to meet the requests or requirements of women with disability but not taking any action to (re)solve the situation. In previous studies this had been identified as appointing people with disability into “precarious” roles without the necessary opportunities, resources, and support to succeed (Wilson-Kovacs et al., 2008). Second, surface acting occurred when women with disability deliberately managed the emotions of colleagues who, either intentionally or without thought placed these women in the “otherness” category. The women with disability described how they managed others' perceptions of their disability, by “disappearing” their disability (Boucher, 2017) in order to fit into the workplace. While surface acting is associated with negative connotations, in the context of our research our interview participants indicated that this approach sometimes elicited positive outcomes.

Managers and work colleagues who surface-acted (Hochschild, 1993; Mann, 2005) and did not deliver promised changes and adaptions, stimulated the confidence in some women to more forcefully claim their disability identity. In doing so, they took on an advocacy role and pushed for the education of workplace colleagues about the needs of women and people with disability in the organization. Assertiveness was required by these women for reasonable adjustments to occur. On the other hand, other women with disability told us that they felt unsupported when their manager surface-acted and workplace barriers remained unaddressed, in consequence they left the organization.

Both positive and negative experiences in the workplace of the women interviewed, reinforce the benefits of good mentors and support networks. As evident in the findings, mentors and support networks can greatly assist to build the confidence and career progression of women with disability. While several of our interviewees had sourced their own external mentor or support group, the opportunity exists for organizations to also initiate support networks and formalizing mentor programs. While a plethora of generic women's support and leadership programs exist, evidence from para-sport research shows that a tailored approach improves the benefits of online support networks and these can lead to offline mentor connections (Bundon and Clarke, 2015).

### Limitations

The participants were sourced from a defined geographical area (Victoria, Australia), and therefore sport organizations located in other jurisdictions might have either more explicit (or less) legal or institutional requirements to provide accessible workplaces. We also note the fluidity of the social construction of disability, especially in cases where the impairment is not visible. In these instances, unless there is self-disclosure or disability identification, the woman with disability's colleagues and/or managers may not be aware of their associated (in)actions.

It is acknowledged that our paper contains some potentially homogenizing generalizations associated with the categorisation of women with disability. In addition, the categorisation of a singular social grouping that does not recognize broader intersectionality related to multiple social-group identities including ethnicity, LGBTIQ+ and class. The point is not to deny the importance of this categorisation but to focus on their related experience (McCall, 2005). In this case, shifting the attention away from the individuals with impairments onto the problems created by the disabling workplace environments and associated barriers (Barnes, 2012).

### Future Research

While our research was conducted in the particularly nuanced context of sport, that is primarily an able-bodied male dominated sector, findings did not differentiate between this and other workplace sectors. To determine if differentiation exists, longitudinal designs in different national contexts focused on national employment data could be sourced to empirically investigate women employees with disability across sectors related to recruitment, retention and human resource management and sport factors.

The focus for future research could integrate the disabling environments with individuals with disability to identify if different impairments shape women's experiences in a sport context. We recognize the disabling environment could be associated with any sector, and the point is the need to interrogate the sport sector to a greater capacity to investigate if differences between sport and other sectors do occur.

Shifting the research focus to the individual could also be extended and applied from an intersectional perspective on women with disability to intersect with age and/or ethnicity (Jammaers and Williams, 2020). In doing so, research could delve into the sport setting to identify if the disability identity of women differs according to her age and/or ethnicity. There is also an opportunity to expand our research on women with disability and conduct quantitative research from a comparative perspective, to determine if particular strategies are required for specific sport settings or for the varied leadership roles that exist in sport. Given the identified importance of having good mentors and support networks, future studies could explore the role of mentors and mentor networks in disability identity work, and as a source of social capital. To further expand the opportunity to better conceptualize diversity and inclusive work environments, is to delve deeper into intersectionality. Research could profitably explore how people negotiate multiple identities at one time from a range of social groupings including gender, age, class, sexual orientation, ethnicity and disability, to build our knowledge on intersectional sensitive approaches (McBride et al., 2015). Consideration could also be given to the use of participative action research. In this case to build “allyship” for women with disability through constant learning, shifting mindsets, and challenging dominant perceptions (Forber-Pratt et al., 2018).

In conclusion, our research with passionate women who took pride in their disability and managed workplace engagements demonstrated how intersectionality with gender and disability is enacted in the sport workplace environment. It identified how relationship management strategies are implemented to navigate employment and/or voluntary roles, and what interpersonal and organizational factors are recognized to attract and retain these women. The findings provide a starting point to encourage future research to delve deeper in the under-researched field of women with disability in the sport workplace environment.

## Data Availability Statement

The datasets presented in this article are not readily available because confidential data permission gained by participants in this study restricts distribution availability of generated dataset. Requests to access the datasets should be directed to clare.hanlon@vu.edu.au.

## Ethics Statement

The study was reviewed and approved by the Victoria University Human Research Ethics Committee (HRE19-023). The participants provided their written or verbal informed consent to participate in this study.

## Author Contributions

CH contributed to conception and design of the study. CH and TT organized the database, analyzed data, and wrote the first draft of the manuscript. Both authors contributed to manuscript revision, read, and approved the submitted version.

## Conflict of Interest

The authors declare that the research was conducted in the absence of any commercial or financial relationships that could be construed as a potential conflict of interest.

## Publisher's Note

All claims expressed in this article are solely those of the authors and do not necessarily represent those of their affiliated organizations, or those of the publisher, the editors and the reviewers. Any product that may be evaluated in this article, or claim that may be made by its manufacturer, is not guaranteed or endorsed by the publisher.
